# Automated de-identification of clinical free-text

**DOI:** 10.23889/ijpds.v1i1.345

**Published:** 2017-04-19

**Authors:** Azad Dehghan, Tom Liptrot, Catherine O'hara, Matthew Barker-Hewitt, Daniel Tibble, Goran Nenadic

**Affiliations:** 1 The Christie NHS Foundation Trust; 2 The University of Manchester

## Objectives

Increasing interest to use unstructured electronic patient records for research has attracted attention to automated de-identification methods to conduct large scale removal of Personal Identifiable Information (PII). PII mainly include identifiable information such as person names, dates (e.g., date of birth), reference numbers (e.g., hospital number, NHS number), locations (e.g., hospital names, addresses), contacts (e.g., telephone, e-mail), occupation, age, and other identity information (ethnical, religion, sexual) mentioned in a private context.

De-identification of clinical free-text remains crucial to enable large-scale data access for health research while adhering to legal (Data Protection Act 1998) and ethical obligations. Here we present a computational method developed to automatically remove PII from clinical text.

## Approach

In order to automatically identify PII in clinical text, we have developed and validated a Natural Language Processing (NLP) method which combine knowledge- (lexical dictionaries and rules) and data-driven (linear-chain conditional random fields) techniques. In addition, we have designed a novel two-pass recognition approach that uses the output of the initial pass to create patient-level and run-time dictionaries used to identify PII mentions that lack specific contextual clues considered by the initial entity extraction modules. The labelled data used to model and validate our techniques were generated using six human annotators and two distinct types of free-text from The Christie NHS Foundation Trust: (1) clinical correspondence (400 documents) and (2) clinical notes (1,300 documents).

## Results

The de-identification approach was developed and validated using a 60/40 percent split between the development and test datasets. The preliminary results show that our method achieves 97% and 93% token-level F1-measure on clinical correspondence and clinical notes respectively. In addition, the proposed two-pass recognition method was found particularly effective for longitudinal records. Notably, the performances are comparable to human benchmarks (using inter annotator agreements) of 97% and 90% F1 respectively.

## Conclusions

We have developed and validated a state-of-the-art method that matches human benchmarks in identification and removal of PII from free-text clinical records. The method has been further validated across multiple institutions and countries (United States and United Kingdom), where we have identified a notable NLP challenge of cross-dataset adaption and have proposed using active learning methods to address this problem. The algorithm, including an active learning component, will be provided as open source to the healthcare community.

